# Beyond BRCA Status: Clinical Biomarkers May Predict Therapeutic Effects of Olaparib in Platinum-Sensitive Ovarian Cancer Recurrence

**DOI:** 10.3389/fonc.2021.697952

**Published:** 2021-07-29

**Authors:** Kazuho Nakanishi, Takashi Yamada, Gen Ishikawa, Shunji Suzuki

**Affiliations:** ^1^Department of Obstetrics and Gynecology, Nippon Medical School Chiba Hokusoh Hospital, Chiba, Japan; ^2^Department of Obstetrics and Gynecology, Nippon Medical School, Tokyo, Japan

**Keywords:** BRCA mutations, neutrophil-lymphocyte ratio, olaparib, ovarian cancer, systemic inflammation index

## Abstract

The purpose of this study was to investigate the predictors of the effect of olaparib on platinum-sensitive recurrent ovarian cancer with unknown germline BRCA mutations. We retrospectively examined 20 patients with platinum-sensitive ovarian cancer who were treated at the Nippon Medical School Chiba Hokusoh Hospital, Japan, from 2018 to 2020. We found that the median progression-free survival was 11.4 months (95% Confidence interval (CI): 3.8–Not Available (NA)) in the group with NLPN score [recurrent neutrophil-lymphocyte ratio (rNLR) × number of previous regimens] >7.51, and median progression-free survival was not reached in the group with NLPN score <7.51 (95% CI: 21.8–NA) (p = 0.0185). There was a clear correlation between the degree of dose reduction of olaparib and recurrence (p = 0.00249). Our results show that NLPN scores lower than 7.51 are associated with a favorable outcome of olaparib treatment for platinum-sensitive recurrent ovarian cancer. In cases with a high rNLR, it may be necessary to start olaparib treatment as early as possible to obtain low NLPN scores. Our results imply that the effectiveness of olaparib can be determined after recurrence and before platinum treatment begins. As newer drugs for ovarian cancer are developed, the measurement of biomarker levels at the start of treatment for recurrent ovarian cancer, as shown in our study, may provide strong support for cancer treatment protocols.

## Introduction

Epithelial ovarian cancer (EOC) is the fifth leading cause of cancer-related deaths in women (1). Most patients are already at an advanced clinical stage of EOC at the time of diagnosis as the initial symptoms of EOC are unclear. Therefore, the EOC survival rate is relatively short. EOCs are sensitive to chemotherapy and respond well to platinum/taxanes in the early stages of treatment, but the 5-year recurrence rate remains at 60% to 80%. Tumor development and progression can elicit an adaptive immune response, and antitumor immunity has been shown to be significantly correlated with patient prognosis. EOC is not a single entity, and various histological subtypes can develop from the ovarian epithelium, including low-grade serous, endometrial, clear cell, and mucinous ovarian cancers (2). Of these, high-grade serous ovarian cancer (HGSOC) is the most common histological subtype, and accounts for approximately 70% of all ovarian cancer cases and a majority (90%) of the ovarian cancer-related deaths.

Several prognostic factors have been proposed to reliably predict EOC outcomes, including histology, tumor stage, residual lesions after surgery, weight loss, response to chemotherapy, and BRCA1/2 mutation status (3). In fact, HGSOC is often associated with homologous recombination deficiency, whereas the microsatellite instability (MSI) phenotype is responsible for up to 14% of all ovarian endometrial cancers and approximately 10% of all clear-cell ovarian cancers. Gene expression analysis of endometrial ovarian cancer and HGSOC identified four different molecular subtypes—immunoreactive, differentiated, proliferative, and mesenchymal—with significantly different survival times (4). A reanalysis of the Cancer Genome Atlas (TCGA) classification in the Mayo Clinic cohort of HGSOC patients showed that the longest survival rates occurred in patients with immunoreactive subtypes. However, this classification had to be validated, and the authors were unable to define a predictive role for each subtype (5). In this regard, it has been previously reported that the presence of a high number of tumor-infiltrating lymphocytes (TILs) in the tumor-cell islets, the peritumoral stroma, or both is significantly correlated with improved results (6).

In contrast to the strong immune response with respect to the presence of TILs and a better prognosis in some patients, tumor immune escape (a mechanism by which antitumor immunity is effectively neutralized) is considered one of the main reasons for disease progression and treatment failure in others. The coordination of many mediators, including cytokines (interleukin (IL)-10, tumor growth factor (TGF)-β, and prostaglandin E2), membrane-bound ligands (B7-H1), and programmed cell death-1 (PD-1), is required to suppress tumor cells and immunosuppressive T-regulatory cells (Tregs) such as FOXP3^+^CD4^+^ cells. In addition, tumor-associated macrophages (TAMs) are involved in inhibiting the activity of immune-effector cells such as CD4^+^ T cells, CD8^+^ T cells, and natural killer cells in tumor microenvironments (7).

The poly ADP-ribose polymerase inhibitor (PARPi), olaparib, was recently approved by the United States Food and Drug Administration (8) for therapeutic use in heavily pretreated germline BRCA mutation (gBRCAm)-associated ovarian cancer. The reported response rates were ~40% in gBRCAm and 24% in BRCA1/2 wild-type (BRCAwt) ovarian cancer patients (9). The susceptibility of patients with gBRCAm-associated ovarian cancer to DNA-damaging agents, including PARPi, has validated the use of gBRCAm as a predictive biomarker for PARPi response (10). However, at least half the gBRCAm-positive HGSOC women do not respond well to PARPi, whereas many BRCAwt-positive HGSOC women respond well to PARPi. The challenge remains to identify, develop, and validate biomarkers to apply within this gBRCAm-positive HGSOC patient population to predict more accurately who will benefit from PARPi therapies.

Recently, in addition to BRCA, homologous recombination deficiency, platinum sensitivity, excision repair cross complementation group 1 (ERCC1), and ADP-ribosylation (ADPRylation) levels have been reported as factors that predict the therapeutic effect of PARPi; however, there are no reports on predictive biomarkers that can be easily measured (11, 12). The neutrophil-to-lymphocyte ratio (NLR), defined as the ratio of neutrophil count to lymphocyte count, is the systemic potential balance between neutrophil-dependent tumor-promoting inflammation and lymphocyte-related antitumor immunity. This inflammatory biomarker is used most widely to evaluate the prognosis of cancer (13–15). Elevated NLR in EOC patients has been found to be associated with a poor prognosis (16, 17). It has also been confirmed that high NLR correlates with the immune profile of the patient (18). Regardless of all the prognostic factors assessed so far, platinum sensitivity, defined as a patient who experiences a recurrence 6 months after the end of primary platinum-based chemotherapy, is considered a predictor of survival outcome in EOC patients (19).

The purpose of this study was to explore the predictors of the effects of olaparib on platinum-sensitive recurrent ovarian cancer by analyzing data, such as NLR, systemic inflammation index (SII), blood inflammatory response, cancer antigen-125 (CA125) levels, and the number of chemotherapy rounds that the patients have undergone.

## Materials and Methods

### Ethical Approval

Written informed consent was obtained from all patients. In addition, approval was obtained from the Ethics Committee of the Nippon Medical School Chiba Hokusoh Hospital (approval number: 874).

### Patient History

Between January 2018 and December 2020, 28 patients were diagnosed with ovarian cancer recurrence at our hospital. Of these patients, 22 were diagnosed with platinum-sensitive recurrence when the recurrence occurred more than 6 months after the last use of platinum-based anticancer drugs. Of these 22 patients, 20 had chosen olaparib as maintenance therapy after treatment with platinum-based anticancer drugs, none chose maintenance treatment with bevacizumab, one did not wish for maintenance therapy after platinum-based anticancer drugs, and one chose best supportive care as her performance status was poor. The remaining six patients developed platinum-resistant recurrences. Platinum-based drug treatment in the 20 patients selected for this study included Paclitaxel (PTX) (175 mg/m^2^ day 1) + Carboplatin (CBDCA) (area under the concentration-time curve (AUC) 5 day 1) every 21 days, PTX (80 mg/m^2^ day 1, 8, 15) + CBDCA (AUC 6 day 1) every 21 days, Gemcitabine (1000 mg/m^2^ day 1, 8) + CBDCA (AUC 4 day 1) every 21 days, and Irinotecan (60 mg/m^2^ day 1, 8, 15) + Cisplatin (60 mg/m^2^ day 1) every 21 days; these previous treatments lasted from 2 to 4, 4, 4, and 4 months, respectively.

The 20 patients subjected to olaparib maintenance therapy for platinum-sensitive recurrent ovarian cancer were eligible for our study. Doses ranged from 400-600 mg/day and treatment time had a mean of 12.9 months (range 4-30 months). Of these 20 patients, 9 had recurrence after the start of olaparib administration: median time to recurrence 9.9 months (range 4-22 months). No dropouts due to side effects were observed in any case. Olaparib dose reduction was performed in 11 patients. Among them, one patient received a one-step dose reduction for anemia and 10 patients received a two-step dose reduction [eight for renal dysfunction (creatinine clearance <50) and two for persistent fatigue]. The basic dose was 600 mg/day, with a one-step dose reduction to 500 mg/day and a two-step dose reduction to 400 mg/day.

### Study Design

Here, we retrospectively examined 20 patients with platinum-sensitive ovarian cancer who were treated at the Nippon Medical School Chiba Hokusoh Hospital from 2018 to 2020. The patients were placed into two groups: those who relapsed after starting olaparib treatment and those who did not. The age, histology, and number of treatment regimens before recurrence for all patients are shown in **Table 1**. The method proposed by Ledermann et al. was used as the starting standard and dose-reduction standard for olaparib administration (10). The patients were divided into three groups based on olaparib drug reduction: no dose reduction; one-step dose reduction; and two-step dose reduction. The primary endpoint was recurrence-free survival.

**Table 1:** Patient characteristics (n = 20).

	Recurrent-	Recurrent+	P
Number of patients (n)	11	9	
Age; average (years)	61.36 ± 11.79	62.33 ± 9.97	0.847
HGSOC (n)	5	5	1
NPR; average (n)	2.63 (range 2-6)	4 (range 2-7)	0.0527^†^
Therapeutic effect by platinum-based therapy			
CR (n)	9	4	0.172*
PR (n)	2	5	
No dose reduction of olaparib (n)	8	1	0.00249**^†^
One-step dose reduction (n)	1	0	
Two-step dose reduction (n)	2	8	

HGSOC, high-grade serous ovarian cancer; NPR, number of previous regimens; CR, complete response; PR, partial response; ^†^p < 0.1. *calculated by Log-rank test; **calculated using the Cochran-Armitage test; the revised Response Evaluation Criteria in Solid Tumors (RECIST) guidelines (version 1.1) were used to judge the therapeutic effect [complete response (CR) or partial response (PR)].

### Blood Sample Analysis

Blood samples were obtained from the patients: (i) immediately after detection of recurrence and before the start of platinum-based treatment, and (ii) immediately after completion of platinum-based treatment and before the start of olaparib treatment. These blood samples were used to determine the white blood cell, neutrophil, and lymphocyte counts using a multi-item automatic blood cell analyzer (XE-2100, Sysmex, Kobe, Japan). A chemistry autoanalyzer (Hitachi 7700, Hitachi, Ibaraki, Japan) was used to measure the serum lactate dehydrogenase (LDH) levels that were analyzed using a lactate dehydrogenase kit (L-type LD J, FUJIFILM Wako Pure Chemical Corporation, Osaka, Japan) and the c-reactive protein (CRP) levels that were analyzed using an N-assay LA CRP-S D-type assay (Nittobo Medical, Tokyo, Japan) according to the manufacturer’s protocols. The CA125 levels were determined using the ARCHITECT i2000SR immunoassay analyzer (Abbott, Chicago, IL, USA). The NLR, which has already been reported as a prognostic factor in EOC, was calculated using the neutrophil/lymphocyte count, and the SII was calculated as platelet count × neutrophil/lymphocyte count (20). The NLPN score was calculated by multiplying the rNLR by the number of previous drug regimens.

### Histopathology

Surgical tissue specimens obtained during debulking surgery were stained with hematoxylin and eosin to determine the different histological cancer types of the patients. Specimens positive for serous carcinoma were analyzed for p53 overexpression by immunostaining with anti-p53 [DO-7] antibody (GeneTex, Inc. Irvine, CA, USA) to identify those positive for HGSOC. We also analyzed the number of regimens used thus far, and used the revised Response Evaluation Criteria in Solid Tumors (RECIST) guidelines (version 1.1) to judge the therapeutic effect [complete response (CR) or partial response (PR)] in patients after platinum-based treatment.

### Statistical Analysis

The age, histology, number of treatment regimens before recurrence (**Table 1**), and the NLR, SII, LDH, CRP, and CA125 values at the time of recurrence and before the start of olaparib treatment (**Table 2**) in both groups were analyzed using *t*-tests. Log-rank tests were performed to compare the therapeutic effects of the platinum-based treatment as well as the effects of olaparib dose reduction. The Cochran-Armitage test was used to analyze the trend between the degree of dose reduction of olaparib and recurrence after the start of treatment. The prediction of the therapeutic effects of olaparib from blood sampling data was calculated using the receiver operating characteristic (ROC) curve. The effectiveness of the ROC curve was evaluated using the area under the curve (AUC), and the threshold was set at the point where the sum of sensitivity and specificity was maximized on the ROC curve. All statistical analyses were performed using EZR (Saitama Medical Center, Jichi Medical University, Saitama, Japan), which is a graphical user interface for R (The R Foundation for Statistical Computing, Vienna, Austria). More precisely, it is a modified version of the R commander, which is designed to add statistical functions that are frequently used in biostatistics (21).

**Table 2:** Laboratory findings from blood samples obtained from epithelial ovarian cancer patients (n = 20).

	Recurrent-	Recurrent+	P
At recurrence			
NLR	2.12 ± 0.85	3.00 ± 1.26	0.080^†^
SII	537.64 ± 308.63	684.11 ± 376.95	0.352
LDH (U/L)	195.18 ± 37.39	205.20 ± 72.40	0.693
CRP (mg/L)	0.36 ± 0.57	1.33 ± 2.45	0.217
CA125 (U/mL)	143.92 ± 154.55	591.73 ± 850.50	0.102
Before Olaparib			
NLR	1.86 ± 0.86	2.34 ± 0.95	0.254
SII	407.95 ± 249.70	400.09 ± 237.71	0.944
LDH (U/L)	186.10 ± 40.86	203.33 ± 43.87	0.388
CRP (mg/L)	1.07 ± 2.77	0.18 ± 0.23	0.382
CA125 (U/mL)	56.11 ± 79.02	92.21 ± 174.72	0.546

Data a presented as medians and standard deviation. NLR, neutrophil-lymphocyte ratio; SII, Systemic inflammatory index; LDH, lactate dehydrogenase; CRP, c-reactive protein; CA125, cancer antigen-125; ^†^means p < 0.1.

## Results

### Patient Characteristics

Eleven patients did not relapse and nine relapsed. The average age of these patients was 61.36 and 62.33 years, respectively, with the most common histology of each being HGSOC, as shown in **Table 1**. Patients that relapsed had been treated with more previous platinum-based regimens (NPRs) before olaparib than patients (on average 4 regimens) than patients that did not relapse (on average 2.63 regimens). Immunohistochemical studies revealed overexpression of p53 in 6 of 20 cases, 4 of which were negative p53 and 10 of which were not available. These cases with p53 overexpression are not associated with recurrence (Odds ratio 1 95% CI 0.0422–23.671).

### Platinum-Based Therapy and Relapse Data

The average progression-free survival (PFS) during the observation period was 12.9 months. The application of the revised RECIST guidelines (version 1.1) after platinum-based treatment showed 13 cases of CR and 7 cases of PR (**Table 1**) (22). Complete response was lower in patients with recurrence and PR was higher, but these differences were not statistically significant from those for patients without recurrence. Olaparib dose reduction was far more frequent in the group that had recurrence (8/11; 73%; versus 1/9, 11% in patients with no recurrence; p = 0.00249).

### Laboratory Findings and Olaparib Effectiveness

Regarding bloodwork and olaparib effectiveness (**Table 2**), we investigated the relationship of neutrophil to lymphocyte counts (NRL). The Pearson product-moment correlation coefficient for NLR at recurrence (rNLR) and NLR before olaparib was 0.473 (95% CI 0.0383–0.757, p = 0.0353).

### NLPN Findings

**Figure 1:**
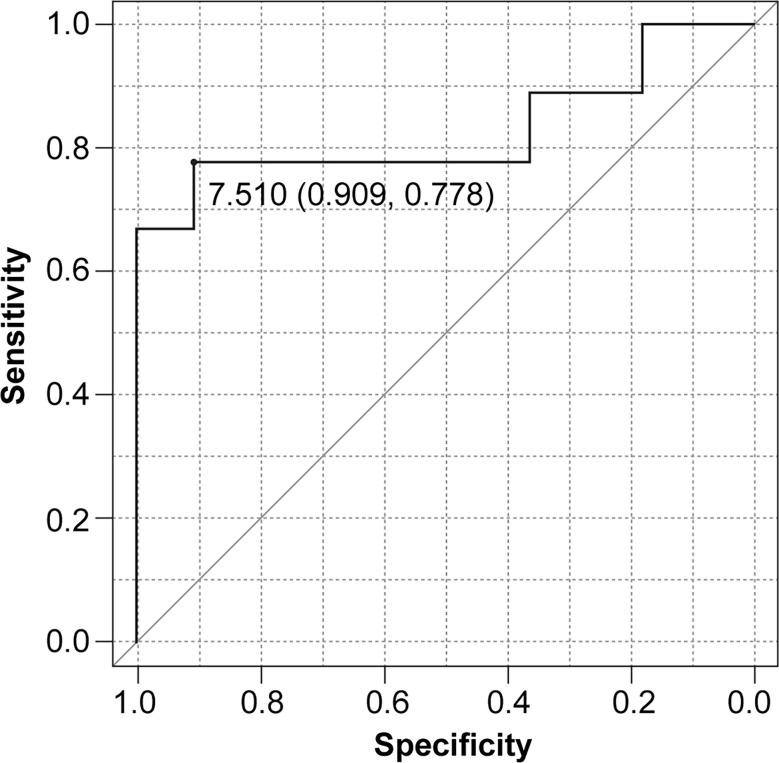
Receiver operating characteristic (ROC) curve for progression-free survival (PFS) based on the NLPN score (neutrophil-lymphocyte ratio at recurrence × number of previous regimens followed).

When combining rNLR and NPR data, we obtained NLPN scores. The AUC of the ROC curve for PFS was 0.828 (95% CI 0.611–1), and the threshold NLPN score value NPR at which the sum of sensitivity and specificity is maximized on the ROC curve was 7.51 (sensitivity = 0.909, specificity = 0.778) (**Figure 1**).

**Figure 2:**
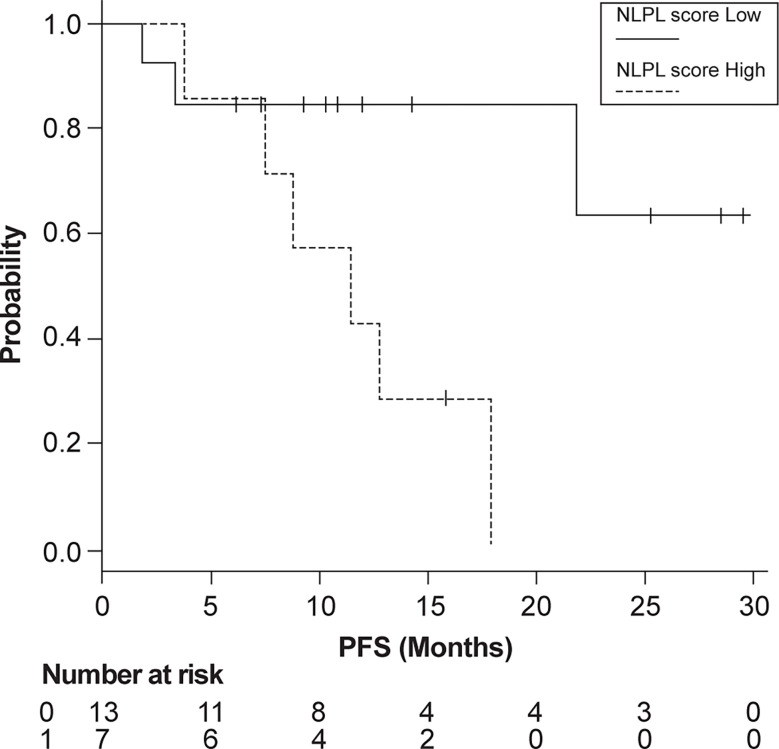
Progression-free survival (PFS) in patients grouped according to high (>7.51) and low (<7.51) NLPN scores (neutrophil-lymphocyte ratio at recurrence × number of previous regimens followed).

Patients were then grouped according to high (>7.51) and low (<7.51) NLPN scores. The median PFS was 11.4 months in the high-NLPN score group (95% CI 3.8–NA). The median PFS was not reached in the low-NLPN score group (95% CI 21.8–NA, p = 0.0185) (**Figure 2**).

There was a clear correlation between the degree of olaparib dose reduction and recurrence (p=0.00249) (**Table 1**), and no correlation between the NLPN score and dose reduction (p = 0.202) (**Table 3**). As the aim of our study was to find predictors of therapeutic effect at the stage of recurrence, we did not use dose reduction after the start of treatment as a predictor.

**Table 3:** Relationship between olaparib dose reduction and NLPN (neutrophil-lymphocyte ratio at recurrence × previous number of regimens) score.

	NLPN score >7.51	NLPN score <7.51
No dose reduction (600 mg/day)	2	7
One-step reduction (500 mg/day)	0	1
Two-step reduction (400 mg/day)	5	5

Cochran-Armitage test p = 0.202.

## Discussion

We identified two important clinical issues in this study.

### Low NLPN Scores Are Associated With Favorable Outcomes in Patients With Platinum-Sensitive Recurrent Ovarian Cancer

We investigated the inflammation index as a predictor of olaparib susceptibility in patients with platinum-sensitive recurrent EOC and the potential value of the number of treatment regimens they have received. We found that a low NLPN score was an independent predictor of platinum sensitivity when adjusted for age, histology, and platinum-treatment response (CR or PR).

The NLR just before starting olaparib after the end of platinum treatment also predicted the therapeutic effect of olaparib. However, there was a correlation between the rNLR and the NLR just before starting olaparib administration. This implies that the effectiveness of olaparib is determined and measurable after recurrence and before platinum treatment begins.

The role of neutrophils in promoting ovarian cancer has been previously described (23), as well as the predictive role of inflammation on tumor progression and treatment success (24). The association of NLR with cancer progression has been found in many studies. The NLPN is an index that improves NLR by adding information on drug susceptibility at the beginning of the maintenance treatment. As new drugs for ovarian cancer are being developed continuously, the measurement of biomarkers at the start of treatment for recurrent ovarian cancer, as shown in our study, may provide strong support in cancer treatment protocols.

### Degree of Olaparib Dose Reduction Is Significantly Correlated to Disease Recurrence

There was no correlation between the NLPN score and the dose of olaparib (Cochran-Armitage test, p = 0.202). To date, no reports have been associated with olaparib and platinum-sensitive recurrent ovarian cancer. In a previous clinical trial that showed the effect of maintenance treatment with olaparib on platinum-sensitive recurrent ovarian cancer, the initial dose was 400 mg; therefore, the two-step dose reduction of 400 mg in our study was not too small, as the therapeutic effect of the 400 mg dose was insufficient (25). Rather, the default dose of 600 mg in our study, which could be considered an overdose compared to the 400 mg dose determined in the trial, may be considered more therapeutic. The therapeutic effect of olaparib was directly correlated with the drug dose after the start of treatment. As the main purpose of this study was to identify biomarkers for olaparib at the time of recurrence, the dose of olaparib was not included in the analysis of therapeutic efficacy predictors.

The dose setting for olaparib in Study 19 is based on the Phase 1 study presented at ASCO 2007 (10, 26). On the other hand, the SOLO2 trial was designed to positively confirm the findings made in Study 19 in a similar disease setting. An adaptive-design phase 1 trial of olaparib bioavailability (Study 24; NCT00777582) (27) has previously established that olaparib exposure with a 300 mg twice-daily tablet dose was similar to, or higher than, exposure in patients receiving olaparib 400 mg twice-daily capsule (10). Therefore, our study complements Study 24, and the experience must be carried on to the more relevant early settings.

Currently, the only PARPi effectiveness biomarkers are BRCA, homologous recombination status, and platinum sensitivity (28–30). The insurance approval for the use of olaparib in platinum-sensitive recurrent ovarian cancer in Japan occurred on January 19, 2018, before the gBRCA test had been approved for insurance. Therefore, many of the patients included in this study had an unknown gBRCA status at the time that our study was conducted. In addition, basic experiments suggest the effectiveness of PARPi on tumors with mutations in both BRCA2 and p53 (31). Since our study was a retrospective study, only 10 of 20 cases could be confirmed by immunohistochemical studies for the presence or absence of p53 overexpression: No correlation was found between p53 overexpression and recurrence after olaparib. This may be one of the main limitations of our study. Another limitation is the retrospective nature of the survey, which may have biased the data analysis. However, the consistency of the results produced by similar previous studies suggests that inflammatory indicators should be tested as prognostic and predictive markers.

Another limitation of our study was the small patient sample size that can cause bias in interpreting the relationship between NLPN scores and clinical responses. Additionally, this post-hoc study of 20 patients was for exploratory purposes, and the sample size of the study was insufficient to effectively address this relationship in detail. Therefore, the NLPN score may not be able to predict clinical response. As this was a retrospective study, the association between high and low NLPN scores and gBRCA1/2 status was unknown.

## Conclusions

In conclusion, our results suggest that the NLPN score is an independent predictor of olaparib susceptibility in platinum-sensitive ovarian cancer. A high NLPN score is a poor prognostic factor for olaparib treatment in platinum-sensitive ovarian cancer, regardless of age, histology, or degree of response to platinum treatment (CR or PR). There was a significant correlation between the degree of olaparib dose reduction and recurrence. Since this result supports the conclusions of Study 24, the experience must be carried on to the more relevant early settings. The exploratory nature of our research requires that these findings be investigated in a positive and powerful setting before drawing decisive conclusions. A low NLPN score is associated with a favorable outcome of olaparib treatment for platinum-sensitive recurrent ovarian cancer. In cases with a high rNLR, it may be necessary to start olaparib as early as possible to achieve a low NLPN score.

## Data Availability Statement

The raw data supporting the conclusions of this article will be made available by the authors, without undue reservation.

## Ethics Statement

The studies involving human participants were reviewed and approved by the Ethics Committee of the Nippon Medical School Chiba Hokusoh Hospital. The patients/participants provided their written informed consent to participate in this study.

## Author Contributions

TY and GI contributed to conception and design of the study. KN organized the database, performed experiments, and wrote the manuscript. SS approved the final draft of the article. All authors contributed to the article and approved the submitted version.

## Conflict of Interest

The authors declare that the research was conducted in the absence of any commercial or financial relationships that could be construed as a potential conflict of interest.

## Publisher’s Note

All claims expressed in this article are solely those of the authors and do not necessarily represent those of their affiliated organizations, or those of the publisher, the editors and the reviewers. Any product that may be evaluated in this article, or claim that may be made by its manufacturer, is not guaranteed or endorsed by the publisher.
